# The genome sequence of the common limpet, *Patella vulgata *(Linnaeus, 1758)

**DOI:** 10.12688/wellcomeopenres.20008.1

**Published:** 2023-09-21

**Authors:** Stephen J. Hawkins, Nova Mieszkowska, Rob Mrowicki, Marine Biological Association Genome Acquisition Lab, Darwin Tree of Life Barcoding collective, Wellcome Sanger Institute Tree of Life programme, Wellcome Sanger Institute Scientific Operations: DNA Pipelines collective, Tree of Life Core Informatics collective, Darwin Tree of Life Consortium

**Affiliations:** 1 The Marine Biological Association, Plymouth, England, UK

**Keywords:** Patella vulgata, common limpet, genome sequence, chromosomal, Patellogastropoda

## Abstract

We present a genome assembly from an individual *Patella vulgata* (the common limpet; Mollusca; Gastropoda; Patellogastropoda; Patellidae). The genome sequence is 695.4 megabases in span. Most of the assembly is scaffolded into 9 chromosomal pseudomolecules. The mitochondrial genome has also been assembled and is 14.93 kilobases in length. Gene annotation of this assembly on Ensembl identified 19,378 protein coding genes.

## Species taxonomy

Eukaryota; Metazoa; Eumetazoa; Bilateria; Protostomia; Spiralia; Lophotrochozoa; Mollusca; Gastropoda; Patellogastropoda; Patelloidea; Patellidae; *Patella*; *Patella vulgata* (Linnaeus, 1758) (NCBI:txid6465).

## Background

*Patella vulgata* was first described by Linnaeus in 1758, and unlike the rest of the Patellidae, its taxonomic status has remained remarkably stable. *Patella vulgata* occurs from the Algarve in Portugal to the Artic Circle in Norway ( Southward *et al.*, 1995). It is present in the Faeroe Islands, but absent from Iceland. It is most abundant on more exposed rocky shores, extending into sheltered fucoid dominated shores, where it becomes less abundant. On northern European shores it is the dominant limpet between the tide marks on sheltered to exposed shores. It gives way to *Patella depressa* on the mid and upper shores in southwest Britain and becomes increasingly rare further south in Europe. Lower down on exposed shores and in rockpools it gives way to *Patella ulysipponensis*.

*P. vulgata* is a protandrous hermaphrodite, first maturing as a male and later in life, individuals usually become female ( Orton *et al.*, 1956), although some may remain male all their lives. In addition, some females may revert to being male ( Le Quesne & Hawkins, 2006). There is evidence that sex change is density dependent: when large females were selectively removed from populations simulation human predation, not only did onset of sexual maturity to become males occur at smaller sizes, nut progression from males to females also occurred at smaller sizes ( Borges *et al.*, 2015). It is a simple brood autumn-winter spawner ( Bowman, 1977; Orton *et al.*, 1956).

*P. vulgata* has a larval life of 5 to 14 days, with a feeding veliger stage ( Dodd, 1956; Hodgson *et al.*, 2007). Grazing by *P. vulgata* prevents recruitment of algae in the intertidal on moderately exposed and exposed shores ( Hawkins *et al.*, 2019; Hawkins & Hartnoll, 1983), in particular, preventing stands of fucoid algae developing. This was dramatically demonstrated following excessive application of toxic dispersants to clean up the Torrey Canyon oil spill in 1967. Vast numbers of limpets were killed on shores from Trevone to the Lizard on the Cornish coastline ( Southward & Southward, 1978), leading to massive blooms of green and subsequently fucoid algae. Recovery occurred in a series of damped oscillations on the shores suffering the most from dispersant application, taking up to 15 years to recover ( Hawkins *et al.*, 2017; Hawkins & Southward, 1992).

It can be considered a keystone species in the Northeast Atlantic. In recent years recruitment failure has been noted in southwest England ( Moore *et al.*, 2011) and it has decreased in relative abundance to *P. depressa* on the mid and upper zones of moderately exposed and exposed shores in response to climate warming ( Hawkins *et al.*, 2008; Hawkins *et al.*, 2009).

## Genome sequence report

**Figure 1.**
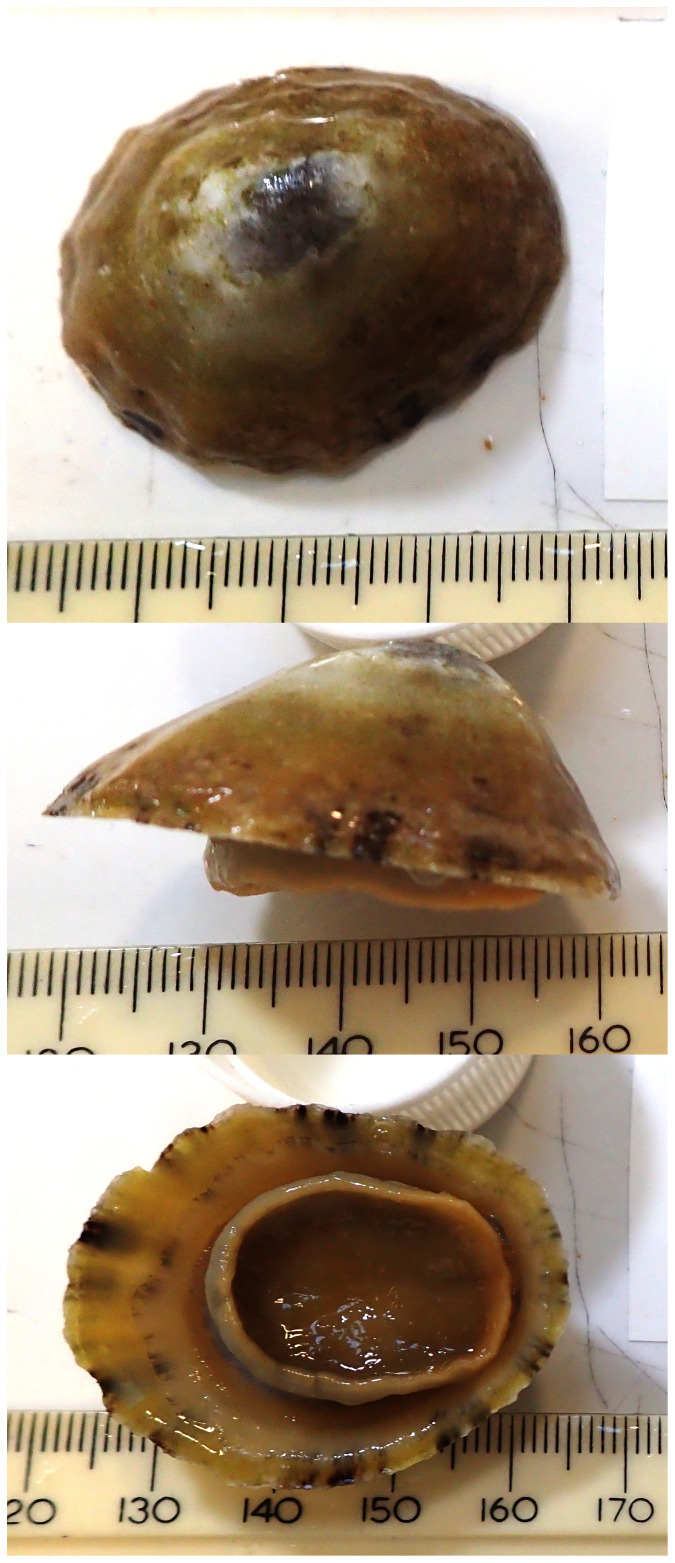
Photographs of the *Patella vulgata* (xgPatVulg1) specimen used for genome sequencing.

The genome was sequenced from a *Patella vulgata* specimen ( Figure 1) collected from Godrevy, Cornwall, UK (50.24, –5.40). A total of 62-fold coverage in Pacific Biosciences single-molecule HiFi long reads and 24-fold coverage in 10X Genomics read clouds were generated. Primary assembly contigs were scaffolded with chromosome conformation Hi-C data. Manual assembly curation corrected 5 missing joins or mis-joins and removed one haplotypic duplication, reducing the scaffold number by 11.54%.

**Figure 2.**
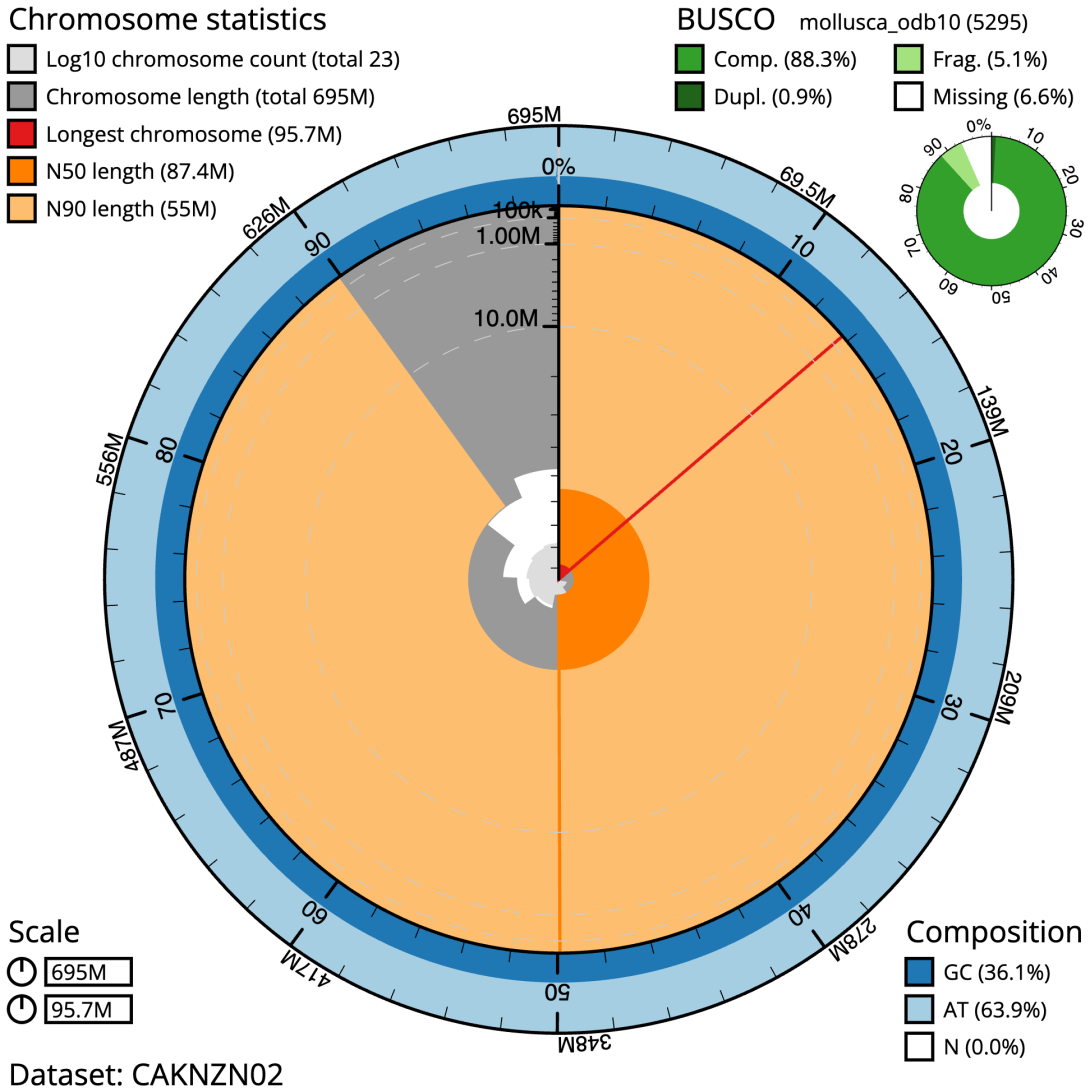
Genome assembly of *Patella vulgata*, xgPatVulg1.2: metrics. The BlobToolKit Snailplot shows N50 metrics and BUSCO gene completeness. The main plot is divided into 1,000 size-ordered bins around the circumference with each bin representing 0.1% of the 695,382,709 bp assembly. The distribution of scaffold lengths is shown in dark grey with the plot radius scaled to the longest scaffold present in the assembly (95,737,551 bp, shown in red). Orange and pale-orange arcs show the N50 and N90 scaffold lengths (87,393,929 and 54,963,775 bp), respectively. The pale grey spiral shows the cumulative scaffold count on a log scale with white scale lines showing successive orders of magnitude. The blue and pale-blue area around the outside of the plot shows the distribution of GC, AT and N percentages in the same bins as the inner plot. A summary of complete, fragmented, duplicated and missing BUSCO genes in the mollusca_odb10 set is shown in the top right. An interactive version of this figure is available at https://blobtoolkit.genomehubs.org/view/xgPatVulg1.2/dataset/CAKNZN02/snail.

The final assembly has a total length of 695.4 Mb in 22 sequence scaffolds with a scaffold N50 of 87.4 Mb ( Table 1). Most (99.96%) of the assembly sequence was assigned to 9 chromosomal-level scaffolds. Chromosome-scale scaffolds confirmed by the Hi-C data are named in order of size ( Figure 2– Figure 5; Table 2). While not fully phased, the assembly deposited is of one haplotype. Contigs corresponding to the second haplotype have also been deposited. The mitochondrial genome was also assembled and can be found as a contig within the multifasta file of the genome submission.

**Table 1.** Genome data for *Patella vulgata*, xgPatVulg1.2.

Project accession data		
Assembly identifier	xgPatVulg1.2	
Species	*Patella vulgata*	
Specimen	xgPatVulg1	
NCBI taxonomy ID	6465	
BioProject	PRJEB50732	
BioSample ID	SAMEA7536243	
Isolate information	xgPatVulg1: muscle (DNA sequencing, Hi-C scaffolding and RNA sequencing)	
Assembly metrics *		*Benchmark*
Consensus quality (QV)	56.3	*≥ 50*
*k*-mer completeness	99.99%	*≥ 95%*
BUSCO **	C:88.3%[S:87.3%,D:0.9%], F:5.1%,M:6.6%,n:5,295	*C ≥ 95%*
Percentage of assembly mapped to chromosomes	99.96%	*≥ 95%*
Sex chromosomes	-	*localised homologous pairs*
Organelles	Mitochondrial genome assembled	*complete single alleles*
Raw data accessions		
PacificBiosciences SEQUEL II	ERR8575363, ERR8575365, ERR8575362, ERR8575364	
10X Genomics Illumina	ERR8571642, ERR8571643, ERR8571641, ERR8571644	
Hi-C Illumina	ERR8571640	
PolyA RNA-Seq Illumina	ERR10378002	
Genome assembly		
Assembly accession	GCA_932274485.2	
*Accession of alternate haplotype*	GCA_932274405.2	
Span (Mb)	695.4	
Number of contigs	67	
Contig N50 length (Mb)	23.8	
Number of scaffolds	22	
Scaffold N50 length (Mb)	87.4	
Longest scaffold (Mb)	95.8	
Genome annotation of assembly GCA_932274485.1		
Number of protein-coding genes	19,378	
Number of non-coding genes	14,639	
Number of gene transcripts	47,283	

* Assembly metric benchmarks are adapted from column VGP-2020 of “Table 1: Proposed standards and metrics for defining genome assembly quality” from ( Rhie *et al.*, 2021). ** BUSCO scores based on the mollusca_odb10 BUSCO set using v5.3.2. C = complete [S = single copy, D = duplicated], F = fragmented, M = missing, n = number of orthologues in comparison. A full set of BUSCO scores is available at https://blobtoolkit.genomehubs.org/view/xgPatVulg1.2/dataset/CAKNZN02/busco.

**Figure 3.**
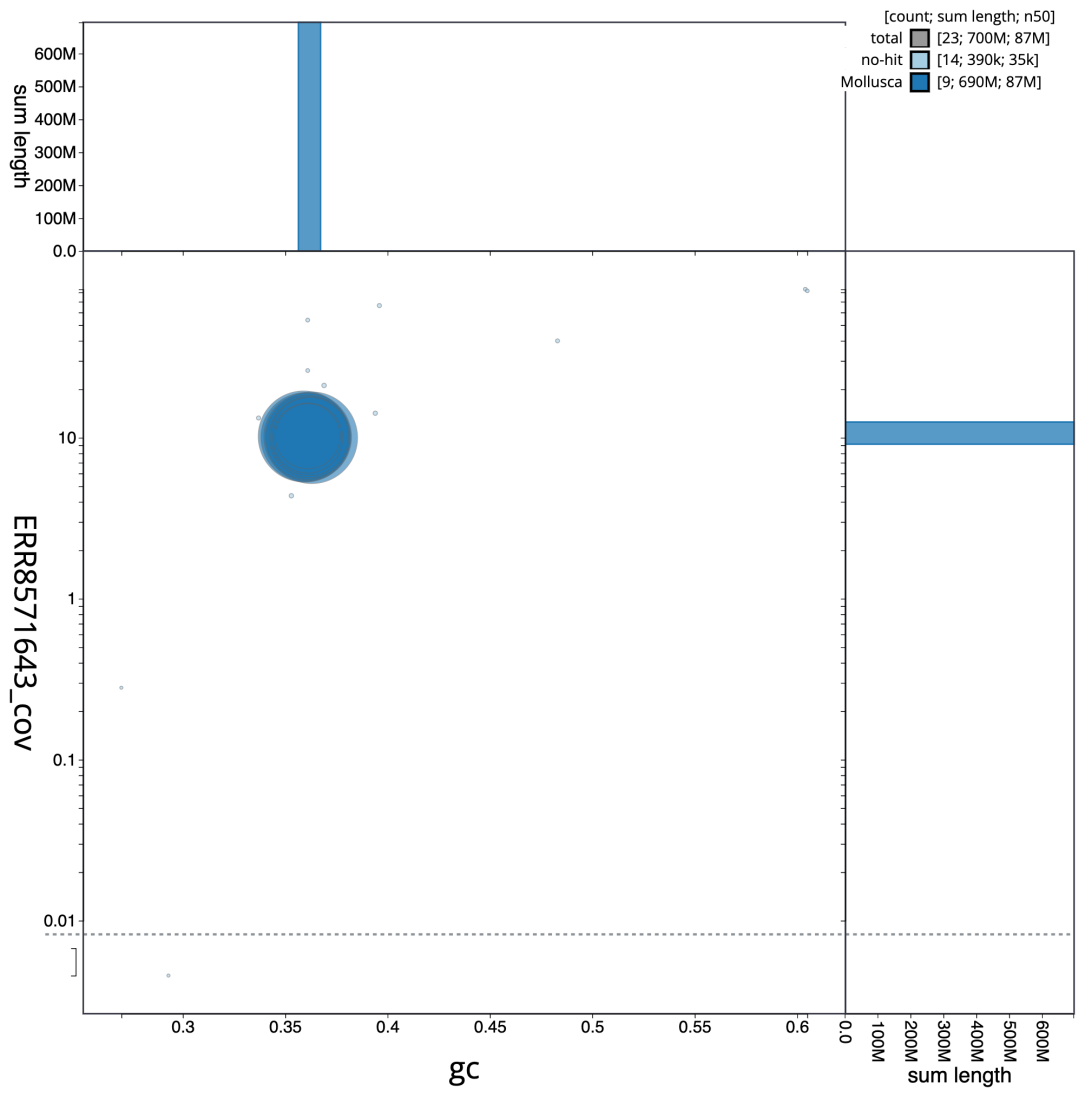
Genome assembly of *Patella vulgata*, xgPatVulg1.2: BlobToolKit GC-coverage plot. Scaffolds are coloured by phylum. Circles are sized in proportion to scaffold length. Histograms show the distribution of scaffold length sum along each axis. An interactive version of this figure is available at https://blobtoolkit.genomehubs.org/view/xgPatVulg1.2/dataset/CAKNZN02/blob.

**Figure 4.**
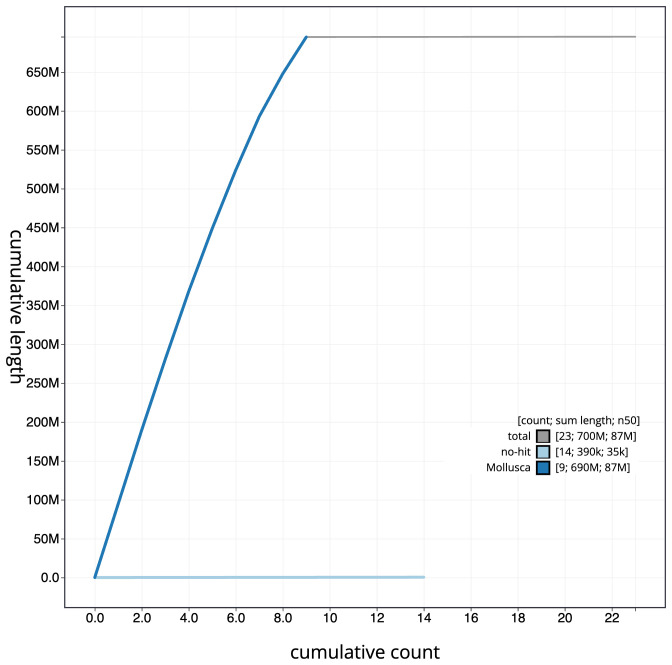
Genome assembly of *Patella vulgata*, xgPatVulg1.2: BlobToolKit cumulative sequence plot. The grey line shows cumulative length for all scaffolds. Coloured lines show cumulative lengths of scaffolds assigned to each phylum using the buscogenes taxrule. An interactive version of this figure is available at https://blobtoolkit.genomehubs.org/view/xgPatVulg1.2/dataset/CAKNZN02/cumulative.

**Figure 5.**
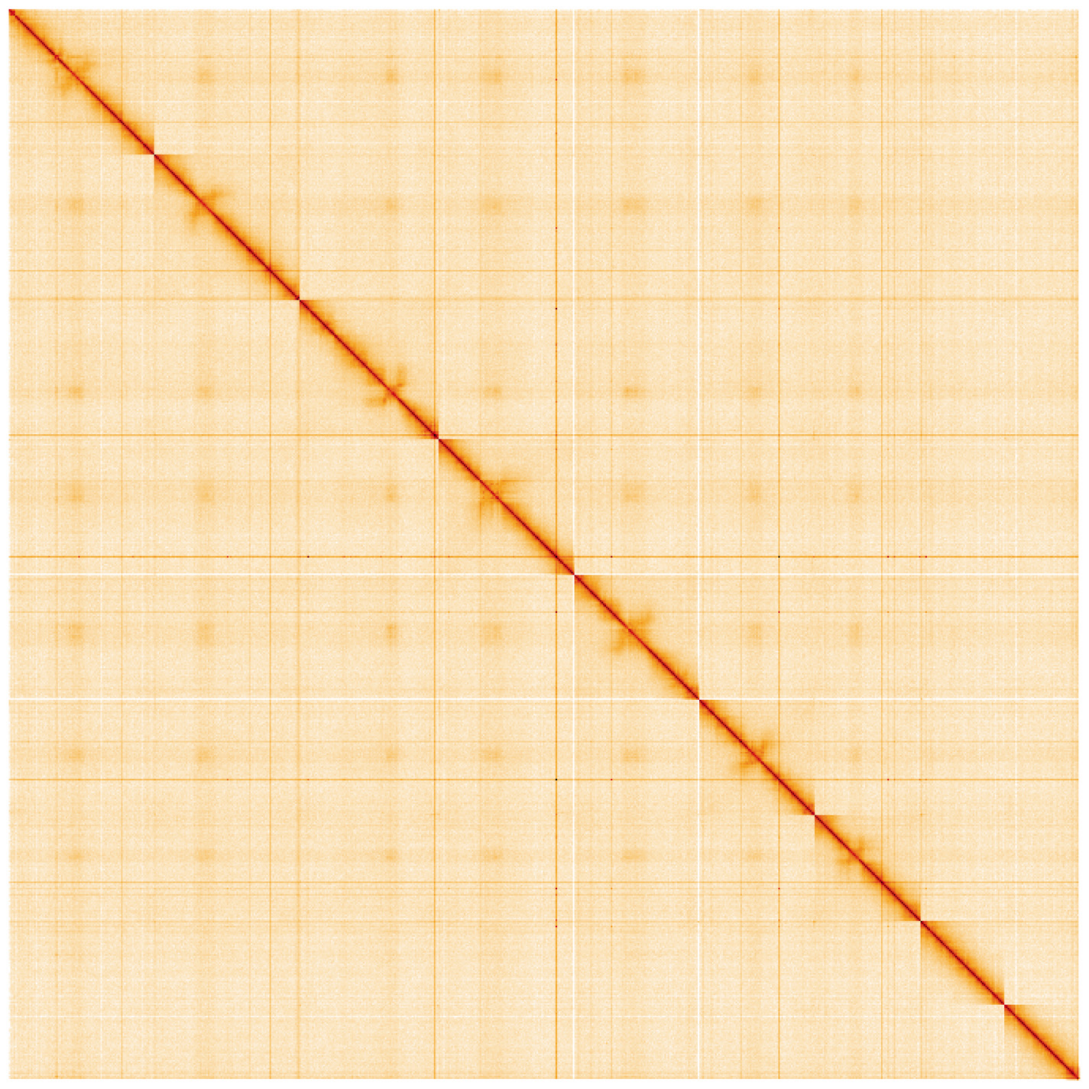
Genome assembly of *Patella vulgata*, xgPatVulg1.2: Hi-C contact map of the xgPatVulg1.2 assembly, visualised using HiGlass. Chromosomes are shown in order of size from left to right and top to bottom. An interactive version of this figure may be viewed at https://genome-note-higlass.tol.sanger.ac.uk/l/?d=Bn4_scjASu-Z8fV43PKCaw.

**Table 2.** Chromosomal pseudomolecules in the genome assembly of *Patella vulgata*, xgPatVulg1.

INSDC accession	Chromosome	Length (Mb)	GC%
OW026348.2	1	95.74	36.5
OW026349.2	2	94.03	36.0
OW026350.2	3	90.89	36.0
OW026351.2	4	87.39	36.0
OW026352.2	5	80.77	36.0
OW026353.2	6	75.11	36.0
OW026354.2	7	68.9	36.0
OW026355.2	8	54.96	36.0
OW026356.2	9	47.19	36.0
OW026357.2	MT	0.01	36.0

The estimated Quality Value (QV) of the final assembly is 56.3 with *k*-mer completeness of 99.99%, and the assembly has a BUSCO v5.3.2 completeness of 88.3% (single = 87.3%, duplicated = 0.9%), using the mollusca_odb10 reference set ( *n* = 5,295).

Metadata for specimens, spectral estimates, sequencing runs, contaminants and pre-curation assembly statistics can be found at https://links.tol.sanger.ac.uk/species/6465.

## Genome annotation report

The *Patella vulgata* genome assembly (GCA_932274485.2) was annotated using the Ensembl rapid annotation pipeline ( Table 1; https://rapid.ensembl.org/Patella_vulgata_GCA_932274485.1/Info/Index). The resulting annotation includes 47,283 transcribed mRNAs from 19,378 protein-coding and 14,639 non-coding genes.

## Methods

### Sample acquisition and nucleic acid extraction

A *Patella vulgata* (specimen ID MBA-200706-003A, individual xgPatVulg1) was collected from Godrevy, Cornwall, UK (latitude 50.24, longitude –5.40) on 2020-07-06. The specimen was collected by hand by Nova Mieszkowska and Rob Mrowicki (Marine Biological Association) and placed in a sample bag. The specimen was identified by Nova Mieszkowska and preserved in liquid nitrogen.

DNA was extracted at the Tree of Life laboratory, Wellcome Sanger Institute (WSI). The xgPatVulg1 sample was weighed and dissected on dry ice with tissue set aside for Hi-C sequencing. Muscle tissue was cryogenically disrupted to a fine powder using a Covaris cryoPREP Automated Dry Pulveriser, receiving multiple impacts. High molecular weight (HMW) DNA was extracted using the Qiagen MagAttract HMW DNA extraction kit. Low molecular weight DNA was removed from a 20 ng aliquot of extracted DNA using the 0.8X AMpure XP purification kit prior to 10X Chromium sequencing; a minimum of 50 ng DNA was submitted for 10X sequencing. HMW DNA was sheared into an average fragment size of 12–20 kb in a Megaruptor 3 system with speed setting 30. Sheared DNA was purified by solid-phase reversible immobilisation using AMPure PB beads with a 1.8X ratio of beads to sample to remove the shorter fragments and concentrate the DNA sample. The concentration of the sheared and purified DNA was assessed using a Nanodrop spectrophotometer and Qubit Fluorometer and Qubit dsDNA High Sensitivity Assay kit. Fragment size distribution was evaluated by running the sample on the FemtoPulse system.

RNA was extracted from muscle tissue of xgPatVulg1 in the Tree of Life Laboratory at the WSI using TRIzol, according to the manufacturer’s instructions. RNA was then eluted in 50 μl RNAse-free water and its concentration assessed using a Nanodrop spectrophotometer and Qubit Fluorometer using the Qubit RNA Broad-Range (BR) Assay kit. Analysis of the integrity of the RNA was done using Agilent RNA 6000 Pico Kit and Eukaryotic Total RNA assay.

### Sequencing

Pacific Biosciences HiFi circular consensus and 10X Genomics read cloud DNA sequencing libraries were constructed according to the manufacturers’ instructions. Poly(A) RNA-Seq libraries were constructed using the NEB Ultra II RNA Library Prep kit. DNA and RNA sequencing was performed by the Scientific Operations core at the WSI on Pacific Biosciences SEQUEL II (HiFi), Illumina NovaSeq 6000 (RNA-Seq and 10X) instruments. Hi-C data were also generated from muscle tissue of xgPatVulg1 using the Arima2 kit and sequenced on the Illumina NovaSeq 6000 instrument.

### Genome assembly, curation and evaluation

Assembly was carried out with Hifiasm ( Cheng *et al.*, 2021) and haplotypic duplication was identified and removed with purge_dups ( Guan *et al.*, 2020). One round of polishing was performed by aligning 10X Genomics read data to the assembly with Long Ranger ALIGN, calling variants with FreeBayes ( Garrison & Marth, 2012). The assembly was then scaffolded with Hi-C data ( Rao *et al.*, 2014) using YaHS ( Zhou *et al.*, 2023). The assembly was checked for contamination and corrected using the gEVAL system ( Chow *et al.*, 2016) as described previously ( Howe *et al.*, 2021). Manual curation was performed using gEVAL, HiGlass ( Kerpedjiev *et al.*, 2018) and Pretext ( Harry, 2022). The mitochondrial genome was assembled using MitoHiFi ( Uliano-Silva *et al.*, 2023), which runs MitoFinder ( Allio *et al.*, 2020) or MITOS ( Bernt *et al.*, 2013) and uses these annotations to select the final mitochondrial contig and to ensure the general quality of the sequence.

A Hi-C map for the final assembly was produced using bwa-mem2 ( Vasimuddin *et al.*, 2019) in the Cooler file format ( Abdennur & Mirny, 2020). To assess the assembly metrics, the *k*-mer completeness and QV consensus quality values were calculated in Merqury ( Rhie *et al.*, 2020). This work was done using Nextflow ( Di Tommaso *et al.*, 2017) DSL2 pipelines “sanger-tol/readmapping” ( Surana *et al.*, 2023a) and “sanger-tol/genomenote” ( Surana *et al.*, 2023b). The genome was analysed within the BlobToolKit environment ( Challis *et al.*, 2020) and BUSCO scores ( Manni *et al.*, 2021; Simão *et al.*, 2015) were calculated.

Table 3 contains a list of relevant software tool versions and sources.

**Table 3.** Software tools: versions and sources.

Software tool	Version	Source
BlobToolKit	4.1.7	https://github.com/blobtoolkit/blobtoolkit
BUSCO	5.3.2	https://gitlab.com/ezlab/busco
FreeBayes	1.3.1-17- gaa2ace8	https://github.com/freebayes/freebayes
gEVAL	N/A	https://geval.org.uk/
Hifiasm	0.16.1-r375	https://github.com/chhylp123/hifiasm
HiGlass	1.11.6	https://github.com/higlass/higlass
Long Ranger ALIGN	2.2.2	https://support.10xgenomics.com/genome-exome/software/pipelines/ latest/advanced/other-pipelines
Merqury	MerquryFK	https://github.com/thegenemyers/MERQURY.FK
MitoHiFi	2	https://github.com/marcelauliano/MitoHiFi
PretextView	0.2	https://github.com/wtsi-hpag/PretextView
purge_dups	1.2.3	https://github.com/dfguan/purge_dups
sanger-tol/genomenote	v1.0	https://github.com/sanger-tol/genomenote
sanger-tol/readmapping	1.1.0	https://github.com/sanger-tol/readmapping/tree/1.1.0
YaHS	yahs-1.1.91eebc2	https://github.com/c-zhou/yahs

### Genome annotation

The BRAKER2 pipeline ( Brůna *et al.*, 2021) was used in the default protein mode to generate annotation for the *Patella vulgata* assembly (GCA_932274485.2) in Ensembl Rapid Release.

### Wellcome Sanger Institute – Legal and Governance

The materials that have contributed to this genome note have been supplied by a Darwin Tree of Life Partner. The submission of materials by a Darwin Tree of Life Partner is subject to the **‘Darwin Tree of Life Project Sampling Code of Practice’**, which can be found in full on the Darwin Tree of Life website here. By agreeing with and signing up to the Sampling Code of Practice, the Darwin Tree of Life Partner agrees they will meet the legal and ethical requirements and standards set out within this document in respect of all samples acquired for, and supplied to, the Darwin Tree of Life Project. 

Further, the Wellcome Sanger Institute employs a process whereby due diligence is carried out proportionate to the nature of the materials themselves, and the circumstances under which they have been/are to be collected and provided for use. The purpose of this is to address and mitigate any potential legal and/or ethical implications of receipt and use of the materials as part of the research project, and to ensure that in doing so we align with best practice wherever possible. The overarching areas of consideration are:

•   Ethical review of provenance and sourcing of the material

•   Legality of collection, transfer and use (national and international) 

Each transfer of samples is further undertaken according to a Research Collaboration Agreement or Material Transfer Agreement entered into by the Darwin Tree of Life Partner, Genome Research Limited (operating as the Wellcome Sanger Institute), and in some circumstances other Darwin Tree of Life collaborators.

## Data Availability

European Nucleotide Archive: *Patella vulgata* (common limpet). Accession number PRJEB50732; https://identifiers.org/ena.embl/PRJEB50732. ( Wellcome Sanger Institute, 2022) The genome sequence is released openly for reuse. The *Patella vulgata* genome sequencing initiative is part of the Darwin Tree of Life (DToL) project. All raw sequence data and the assembly have been deposited in INSDC databases. Raw data and assembly accession identifiers are reported in Table 1.

## Author information

Members of the Marine Biological Association Genome Acquisition Lab are listed here: https://doi.org/10.5281/zenodo.4783604.

Members of the Darwin Tree of Life Barcoding collective are listed here: https://doi.org/10.5281/zenodo.4893703.

Members of the Wellcome Sanger Institute Tree of Life programme are listed here: https://doi.org/10.5281/zenodo.4783585.

Members of Wellcome Sanger Institute Scientific Operations: DNA Pipelines collective are listed here: https://doi.org/10.5281/zenodo.4790455.

Members of the Tree of Life Core Informatics collective are listed here: https://doi.org/10.5281/zenodo.5013541.

Members of the Darwin Tree of Life Consortium are listed here: https://doi.org/10.5281/zenodo.4783558.
