# Supplementary material for: The genome sequence of the common limpet, Patella vulgata (Linnaeus, 1758)
Source: Wellcome Open Res. 2023 Sep 21;8:418. [Version 1] doi: 10.12688/wellcomeopenres.20008.1 (PMC10663661; doi:10.12688/wellcomeopenres.20008.1)
Supplement: Peer review history

# Reviewer report: Reviewer response for version 1

**DOI:** 10.21956/wellcomeopenres.22154.r69415

**Published:** 2023-11-30

**Authors:** David Ferrier

**Affiliations:** 1 School of Biology, University of St Andrews, St Andrews, Scotland, UK

**Is the rationale for creating the dataset(s) clearly described?**

 Yes. Besides the need for sequencing this mollusc as part of the Darwin Tree of Life project, there is the added interest of this animal being a keystone intertidal species that is very widespread and a key “engineer” of these ecosystems. 

**Are the protocols appropriate and is the work technically sound?**

 Yes, these various protocols are certainly appropriate and the work is of a high technical standard. 

**Are sufficient details of methods and materials provided to allow replication by others?**

 Yes, these are ell-described and meet accepted standards for these data notes. 

**Are the datasets clearly presented in a useable and accessible format?**

 Yes, the datasets such as the assembly and sequencing data are accessible and useable. 

 Other minor corrections. 

1. The draft genome of *Patella vulgata* has been previously sequenced and published (Kenny et al., Marine Genomics 24 (2015), 139-146). I was surprised that this previous publication was not mentioned, nor any attempts made to put the current assembly into the context of this previous work. 
2. In the background section the sentence starting “There is evidence that sex change is density dependent…” does not make sense and needs rewriting. 

Are sufficient details of methods and materials provided to allow replication by others?

Yes

Is the rationale for creating the dataset(s) clearly described?

Yes

Are the datasets clearly presented in a useable and accessible format?

Yes

Are the protocols appropriate and is the work technically sound?

Yes

Reviewer Expertise:

NA

I confirm that I have read this submission and believe that I have an appropriate level of expertise to confirm that it is of an acceptable scientific standard.

# Reviewer report: Reviewer response for version 1

**DOI:** 10.21956/wellcomeopenres.22154.r69414

**Published:** 2023-11-21

**Authors:** Rafael Zardoya

**Affiliations:** 1 Departamento de Biodiversidad y Biología Evolutiva, Museo Nacional de Ciencias Naturales (MNCN-CSIC), Madrid, Spain

This genome report presents the sequencing, assembling and scaffolding of the 9-chromosome genome of the common limpet. This species is ecologically relevant in the rocky shores of Western Europe and belongs to the earliest divergent lineage in the Gastropoda. The pipeline from the initial sampling of the individual to the final scaffolding and preliminary annotation of the (almost) one-phased genome followed the high standards established by the Wellcome Sanger Institute. As a result, almost no signal of contamination is found and the BUSCO scores are correct for a gastropod. The comparison of this genome with those of *Patella pelucida *(Lawniczak et al. 2022) ^1^ and *Lottia gigantea* (Simakov et al. 2013) ^2^ will be particularly interesting. With regards to the introduction, I find useful citing that the mitogenome of *P. vulgata* was already sequenced (Uribe et al. 2019) ^3^. Also, I suggest passing the last sentence of the second paragraph to the beginning of the third paragraph: Such as most species of patellogastropod limpets, *P. vulgata* is a broadcast spawners,with an autumn-winter spawning period (Bowman, 1977; Orton et al., 1956). Its life cycle includes a pelagic larval phase (with a feeding veliger stage) that last between 5 and 14 days. Finally, I think that the final sentences of this paragraph (“This was dramatically…15 years to recover) are really anecdotic and digressive, and I suggest omitting these.

Are sufficient details of methods and materials provided to allow replication by others?

Yes

Is the rationale for creating the dataset(s) clearly described?

Yes

Are the datasets clearly presented in a useable and accessible format?

Yes

Are the protocols appropriate and is the work technically sound?

Yes

Reviewer Expertise:

phylogenomics, comparative genomics, gastropods

I confirm that I have read this submission and believe that I have an appropriate level of expertise to confirm that it is of an acceptable scientific standard.

## References

[1] : The genome sequence of the blue-rayed limpet, Patella pellucida Linnaeus, 1758. *Wellcome Open Research* .2022;7: 10.12688/wellcomeopenres.17825.1 10.12688/wellcomeopenres.17825.1 36874573PMC9975397

[2] : Insights into bilaterian evolution from three spiralian genomes. *Nature* .2013;493(7433) : 10.1038/nature11696 526-531 10.1038/nature11696 PMC408504623254933

[3] : New patellogastropod mitogenomes help counteracting long-branch attraction in the deep phylogeny of gastropod mollusks. *Molecular Phylogenetics and Evolution* .2019;133: 10.1016/j.ympev.2018.12.019 12-23 10.1016/j.ympev.2018.12.019 30572020

# Reviewer report: Reviewer response for version 1

**DOI:** 10.21956/wellcomeopenres.22154.r69411

**Published:** 2023-11-09

**Authors:** José María Martín-Durán

**Affiliations:** 1 School of Biological and Behavioural Sciences, Queen Mary University of London, London, UK

This genome note reports the chromosome-level assembly of Patella vulgata. This is a keystone species in the North Atlantic shores with important ecological implications and that has served as a model organism in developmental and evolutionary biology. The assembly is of high quality and follows the gold standards established by the Darwin Tree of Life. It will be an important resource for multiple research fields.

Are sufficient details of methods and materials provided to allow replication by others?

Yes

Is the rationale for creating the dataset(s) clearly described?

Yes

Are the datasets clearly presented in a useable and accessible format?

Yes

Are the protocols appropriate and is the work technically sound?

Yes

Reviewer Expertise:

Evolutionary biology, developmental biology, comparative genomics

I confirm that I have read this submission and believe that I have an appropriate level of expertise to confirm that it is of an acceptable scientific standard.
